# Altered rumen microbiome and correlations of the metabolome in heat-stressed dairy cows at different growth stages

**DOI:** 10.1128/spectrum.03312-23

**Published:** 2023-11-16

**Authors:** Lei Feng, Yu Zhang, Wei Liu, Dewei Du, Wenbo Jiang, Zihua Wang, Ning Li, Zhiyong Hu

**Affiliations:** 1 College of Animal Science and Technology, Shandong Agricultural University, Taian, China; Nanjing Agricultural University, Nanjing, China

**Keywords:** Holstein cows, heat stress, multi-omics, metabolome, growth stage

## Abstract

Heat stress is one of the major stressors affecting dairy cow production, which causes great losses in animal husbandry production and economic development. This study combined 16S rDNA sequencing, metagenomic sequencing, and metabolomic analysis to investigate the effects of heat stress on the rumen microbiome and metabolism of cows. To achieve this, 10 cows each of growing heifers, heifers, and lactating cows were selected for sample collection in April and August (a total of 60 cows). Ruminal fluid was collected, filtered through gauze, and immediately transferred to liquid nitrogen prior to metagenomic, 16S rDNA sequencing, and metabolomic analyses. Heat stress increased the abundance of pathogenic bacteria, such as *Treponema_2*, *Sphingobacterium*, and *Streptococcus*, in cattle at all growth stages, with the structure and composition of rumen microbial communities being the most affected by heat stress in growing heifers and the least affected by heat stress in lactating cows. Heat stress led to a significant decrease in carbohydrate metabolic function in growing heifers and heifers, whereas microbial function was the most stable in lactating cows, suggesting that changes in microbial function are not only more conserved than microbial composition but are also affected by age. Heat stress led to significant enrichment of fatty acyl metabolites in growing heifers, heifers, and lactating cows, and the heat stress indicator species *Bacillus*, *Lactococcus*, *Streptococcus*, and *Enterococcus* were significantly associated with fatty acyl compounds in growing heifers and heifers. Therefore, heat stress alters the metabolic status of cows by modulating fatty acid biosynthesis, the PPAR signaling pathway, and arachidonic acid metabolism through the regulation of long-chain fatty acids and other biomarkers in cows at three growth stages.

**IMPORTANCE:**

Heat stress is one of the main causes of economic losses in the dairy industry worldwide; however, the mechanisms associated with the metabolic and microbial changes in heat stress remain unclear. Here, we characterized both the changes in metabolites, rumen microbial communities, and their functional potential indices derived from rumen fluid and serum samples from cows at different growth stages and under different climates. This study highlights that the rumen microbe may be involved in the regulation of lipid metabolism by modulating the fatty acyl metabolites. Under heat stress, the changes in the metabolic status of growing heifers, heifers, and lactating cows were closely related to arachidonic acid metabolism, fatty acid biosynthesis, and energy metabolism. Moreover, this study provides new markers for further research to understand the effects of heat stress on the physiological metabolism of Holstein cows and the time-dependent changes associated with growth stages.

## INTRODUCTION

With global warming and the increased intensification of farming, heat stress has become a key factor affecting human and animal health (1) and even life safety in areas with high seasonal environmental temperatures. In the feeding and management of dairy cows, heat stress has always been an critical issue that affects their health and limits the economic benefits of dairy cows in summer (2, 3). In recent years, global climate change has led to increased atmospheric carbon dioxide release and daily mean temperatures, making heat stress a major challenge in dairy farming. In the temperature comfort zone, a cow can maintain the balance of body temperature without consuming additional energy. Through a series of behavioral, physiological, and metabolic reactions, the body maintains a balance between heat production and heat dissipation to maintain a constant body temperature and achieve the best production state (4). Approximately 15% of the heat generated during the metabolism of cows is distributed throughout the respiratory tract. The respiratory rate is one of the indicators often observed by the occurrence of heat stress in cows. The respiratory rates for evaporation and heat dissipation can be accelerated to reduce the damage caused by heat stress to the body. Heat stress in animal husbandry production and economic development, caused by the summer temperature rise of mild chronic heat stress (5), which is the main reason for cow stress, causes huge losses; therefore, in August, the whole summer chronic heat stress cow sampling was compared with the normal temperature cow metabolism and microbial composition and function to explore the influence of summer heat stress on cow metabolism and physiology.

Nutrient digestion in ruminants is closely related to the rumen. The rumen is a unique digestive organ with a complex dynamic anaerobic ecosystem and a large number of microorganisms (6). Numerous studies have shown that rumen microorganisms interact with the host, affecting the metabolic process of the host and summer heat stress may lead to changes in rumen microbial composition. Some studies have also explored the influence of temperature on the host microbiota, indicating that the rumen microbiota is sensitive to environmental temperature and temperature-induced changes in the composition and diversity of the rumen microbiota, which may have an important impact on the host phenotype and health (7). Several studies have been conducted on the effects of heat stress on microbial diversity, composition, and function. For example, heat stress can affect ruminal fermentation (8), alter the ruminal fermentation mode (9), and affect the composition and quantity of microbial flora in the rumen (3), leading to a decrease in feed efficiency and production performance (10). Zhong et al. (11) found that heat stress had no significant effect on the microbial alpha diversity of goats; however, the abundance of pathogenic bacteria, such as *Erysipelotrichaceae_UCG-004* and *Treponema_2*, increased, which altered the microbial composition of the rumen and affected metabolic function. Among various environmental factors, temperature has a profound impact on the physiology, behavior, and performance of dairy cows (12), rumen microorganisms are sensitive to environmental temperature, and temperature induces changes in the composition and diversity of rumen microbial communities and changes the functional relationship between host and microbiota.

Ruminal microbial community composition and changes are important breakthroughs in understanding the effects of heat stress on dairy farming technology. Currently, studies on the effects of heat stress on Holstein cows are primarily aimed at specific stages. Few studies have examined Holstein cows at different stages; however, the exposure of cows to summer heat stress has long-term effects on their physiology. Therefore, this study included Holstein cows that developed into growing heifers, heifers, and lactating cows. Understanding the complex interplay between the host and microbes during heat stress is the key to developing strategies to maximize ruminant productivity and address these challenges. Therefore, this study compared the effects of heat stress on the rumen microbiota and metabolome of Holstein cows to further explore the mechanisms of heat stress and improve the feeding management of heat stress in Holstein cows.

## RESULTS

### Trait indicators

Currently, the temperature-humidity index (THI) is commonly used to describe the degree of heat stress in dairy cows. THI can be divided into different ranges according to the degree of heat stress. When THI is <68, cows have no risk of heat stress; when THI is between 68 and 74, they are in mild heat stress zones and cows are negatively affected by heat stress and THI > 75 leads to a sharp decline in production performance (13). As previously described (14), growing heifers, heifers, and lactating cows were subjected to heat stress in August.

### Microbial composition and diversity under different temperature treatments

The diversity of rumen microbial communities under heat stress was assessed using 16S rDNA sequencing. Quality control of the 16S rDNA sequencing of growing heifers, heifers, and lactating cows has been previously reported (14).

**Fig 1:**
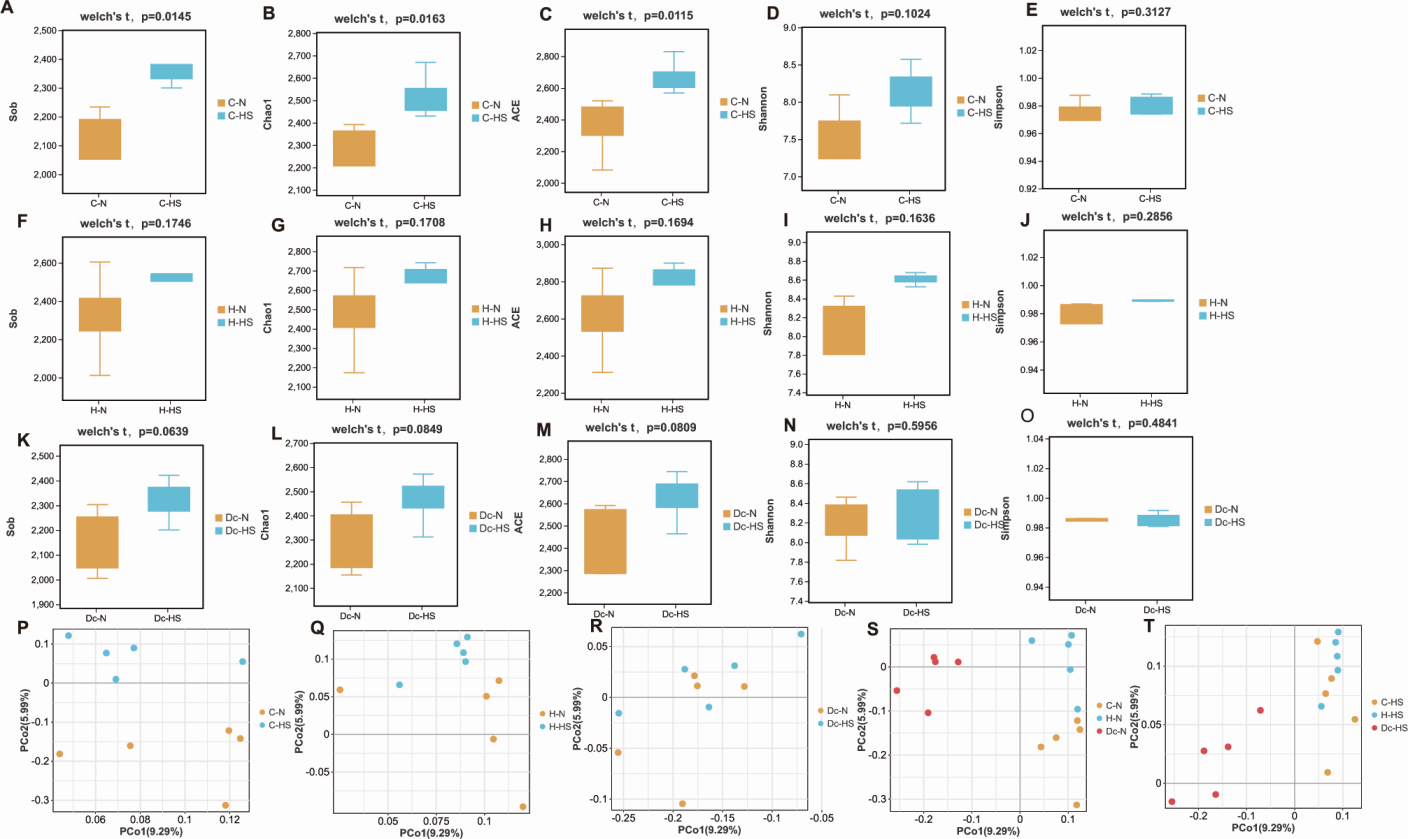
Results of 16S rDNA read sequence of the rumen bacteria in the N and HS groups of growing heifers, heifers, and lactating cows. The Sob, Chao1, ACE, Shannon, and Simpson indices of growing heifers (A–E), heifers (F–J), and lactating cows (K–O); PCoA based on the OTU level of the bacteria in growing heifers (P), heifers (Q), and lactating cows (R); PCoA based on the OTU level of the bacteria in the C-N, H-N, and Dc-N (S); PCoA based on the OTU level of the bacteria in the C-HS, H-HS, and Dc-HS (T). Notes: N and HS represent normal and heat stress groups, respectively; C, H, and Dc represent growing heifers, heifers, and lactating cows, respectively.

In growing heifers, except for Simpson and Shannon, there were significant differences in Sob, ACE, and Chao1 between the C-N and C-HS groups (Fig. 1A through E). There were no significant differences in the above five indicators between the H-N and H-HS groups (Fig. 1F through J) and between the Dc-N and Dc-HS groups (Fig. 1K through O), respectively. Principal coordinate analysis (PCoA) of operational taxonomic unit (OTU) levels with ANOSIM analysis showed significant separation between the C-N and C-HS groups (Fig. 1P; Fig. S1A) and between the H-N and H-HS groups (Fig. 1Q; Fig. S1B), and no significant separations between Dc-N and Dc-HS (Fig. 1R; Fig. S1C). The ruminal microbiota of cows at different growth stages was significantly separated (Fig. 1S and T), and the community structure heterogeneity was significant (*P* < 0.05, ANOSIM analysis), which significantly distinguished the three groups (Fig. S1D and E).

### Different rumen microbial composition associated with heat stress

**Fig 2:**
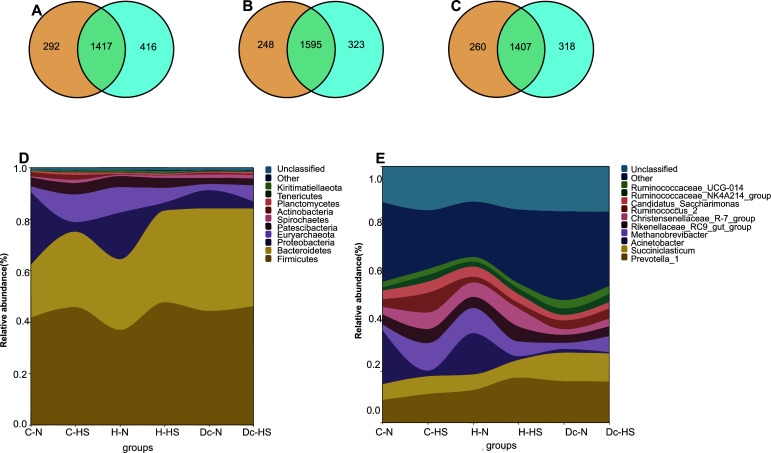
(A–C) Venn diagram illustrating the overlap of rumen microbial OTUs in growing heifers, heifers, and lactating cows. The relative abundance of phylum (**D**) and genus (**E**) of rumen microbiota of dairy cows.

C-HS, H-HS, and Dc-HS had higher OTU numbers than C-N, H-N, and Dc-N (Fig. 2A through C). Through classification, we found that rumen bacteria were dominated by *Firmicutes* and *Bacteroidetes*, whereas the rest were distributed in *Proteobacteria*, *Euryarchaeota*, *Patescibacteria*, *Spirochaetes*, *Actinobacteria*, and others (Fig. 2D). *Prevotella_1*, *Succiniclasticum*, *Acinetobacter*, and *Methanobrevibacte*r were the dominant genera of rumen bacteria, with *Prevotella_1* showing an increase in relative abundance as age advanced (Fig. 2E).

### Indicative species

**Fig 3:**
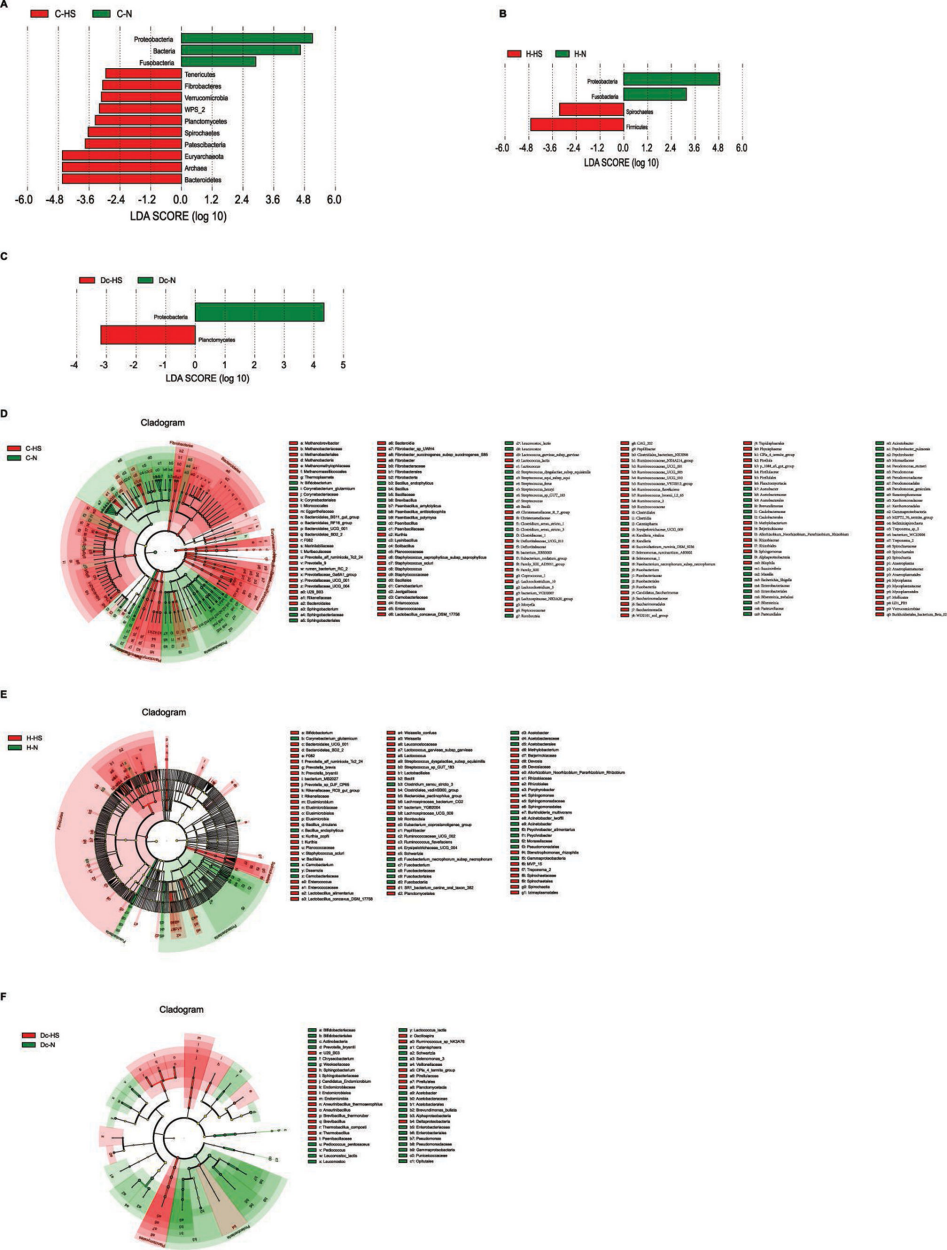
Differential rumen bacterial species between normal and heat stress groups of growing heifers, heifers, and lactating cows. (**A**) Significantly different bacterial species at the phylum level of growing heifers. (**B**) Significantly different bacterial species at the phylum level of heifers. (**C**) Significantly different bacterial species at the phylum level of lactating cows. (**D**) Significantly different bacterial species of growing heifers. (**E**) Significantly different bacterial species of the heifers. (**F**) Significantly different bacterial species of lactating cows. Significant differences were tested via LEfSe analysis, with linear discriminant analysis (LDA) > 2 and *P* < 0.05.

Using linear discriminant analysis effect size (LEfSe) analysis (LDA score> 2), we compared the bacterial taxa in growing heifers, heifers, and lactating cows (Fig. 3). At the phylum level, the microbial level collected from rumen fluid showed that heat stress significantly reduced the relative abundance of *Proteobacteria* and *Fusobacteria* and significantly increased the relative abundance of *Tenericutes*, *Fibrobacteres*, *Verrucomicrobia*, *Planctomycetes*, *Spirochaetes*, *Patescibacteria*, *Euryarchaeota*, and *Bacteroidetes* in growing heifers (Fig. 3A). Heat stress significantly reduced the relative abundance of *Proteobacteria* and *Fusobacteria* and significantly increased the relative abundance of *Spirochaetes* and *Firmicutes* in heifers (Fig. 3B). Heat stress significantly reduced the relative abundance of *Proteobacteria* and significantly increased the relative abundance of *Planctomycetes* in lactating cows (Fig. 3C).

Heat stress increased the relative abundance of *Coprococcus_1*, *Ruminococcus_2*, *Kurthia*, *Treponema_2*, *CPla_4_termite_group*, *Prevotella_9, Moryella*, *Lactococcus*, *Methylobacterium*, *Fibrobacter*, *Enterococcus*, *Methanobrevibacter*, *Sphingomonas*, *Papillibacter*, *Ruminococcaceae*, *Spirochaetia*, *Spirochaetaceae*, *Saccharimonadaceae*, *Fibrobacteraceae*, *Staphylococcaceae*, *Saccharimonadales*, and *Enterococcaceae* in C-HS . *Bacillus*, *Acinetobacter*, *Pseudomonas*, *Streptococcus*, *Bifidobacterium*, *Lysinibacillus*, *Leuconostoc*, and *Stenotrophomonas* were relatively more abundant in the C-N (Fig. 3D).

Heat stress significantly increased the relative abundance of *Treponemae-2, Bifidobacterium, Lactococcus, Kurthia, Papillibacter, Ruminococcaceae_UCG_002, Schwartzia, Methylobacterium, Rikenellaceae_RC9_gut_group, Lachnospiraceae_UCG_009, Enterococcus and Sphingomonas, Leuconostocaceae, Prevotella_bryantii, Bacilli, Streptococcus_sp_GUT_183, Lactobacillus_alimentarius, Rikenellaceae* in H-HS; heat stress resulted in the relative abundance of *Acinetobacter*, *Psychrobacter*, *Acetobacter*, *Fusobacterium*, *Clostridium_sensu_stricto_3*, *Fusobacteriaceae*, *Acetobacteraceae*, *Pseudomonadales*, *Gammaproteobacteria*, *Fusobacteriia,* and *Moraxellaceae* being significantly reduced (Fig. 3E). These microorganisms were more abundant in the H-N.

Heat stress increased the relative abundance of *Candidatus_Endomicrobium*, *Oscillospira*, *CPla_4_termite_group*, *Aneurinibacillus*, *Brevibacillus*, *Aneurinibacillus*, *Sphingobacterium*, *Sphingobacteriaceae*, *Paenibacillaceae*, *Pirellulales*, *Thermobacillus*, *Deltaproteobacteria*, *Endomicrobiaceae*, *Planctomycetacia*, and *Ruminococcus_sp_NK3A7*6 in Dc-HS; *Pseudomonas*, *Acetobacter*, *Selenomonas_3*, *Leuconostoc*, *Pediococcus*, *Chryseobacterium*, *Schwartzia*, *Bifidobacteriaceae*, *Enterobacteriales*, *Enterobacteriaceae*, *Prevotella_bryantii*, *Bifidobacteriaceae*, *Lactococcus_lactis*, *Proteobacteria*, *Acetobacter*, *Alphaproteobacteria*, *Bifidobacteriales*, *Veillonellaceae*, *Gammaproteobacteria*, and *Actinobacteria* were more abundant in the Dc-N (Fig. 3F).

In summary, rumen microbial composition was examined at different temperatures using species composition and indicator species analyses. These results indicate that heat stress significantly changed the rumen microbiota composition of growing heifers, heifers, and lactating cows.

### Functional pathways

Quality control of the metagenomic sequencing of growing heifers, heifers, and lactating cows has been previously reported (14). The function of the rumen microbiome was determined using the Kyoto Encyclopedia of Genes and Genomes (KEGG) map and the genes encoding carbohydrate-active enzymes (CAZymes).

**Fig 4:**
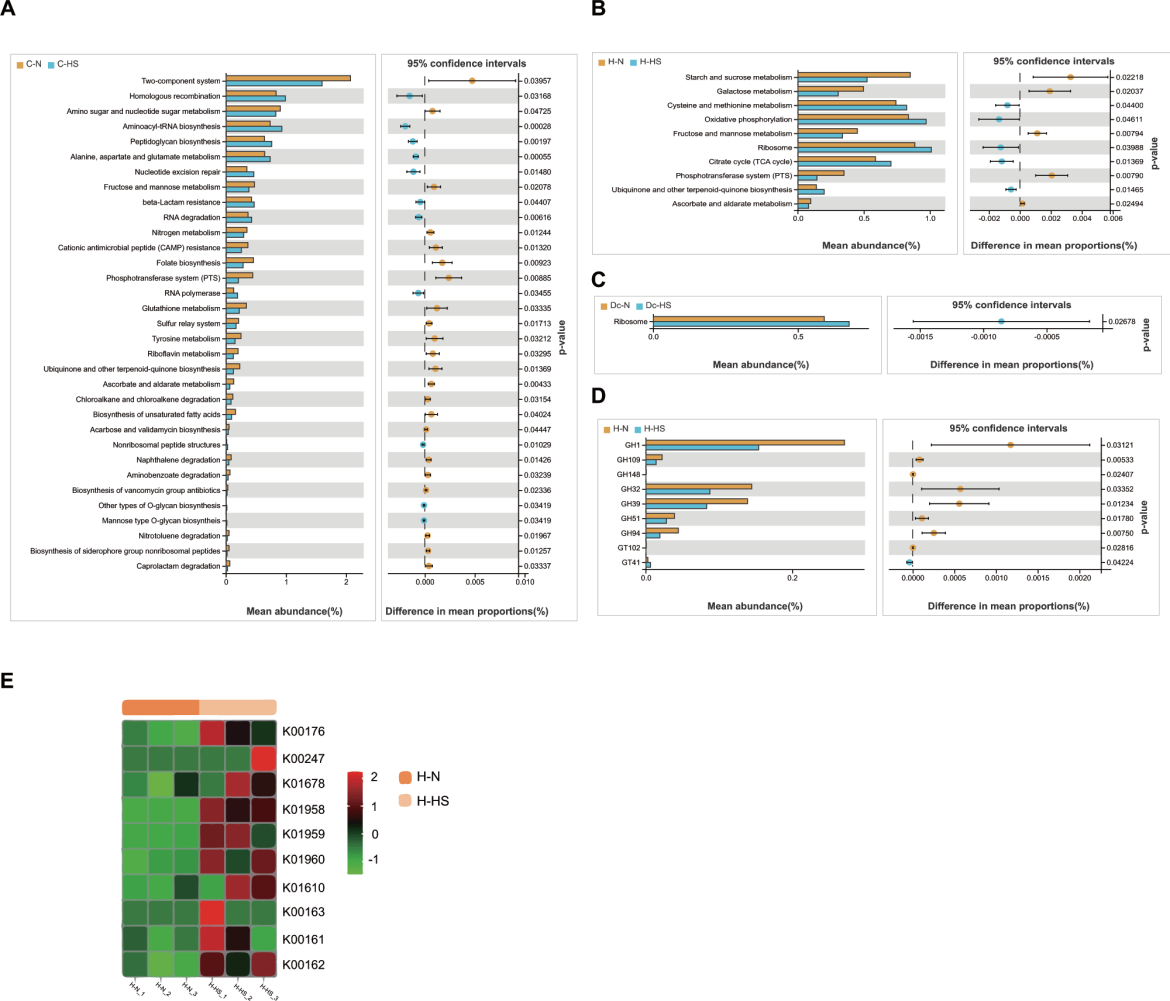
Differential KEGG and CAZyme functions between growing heifers, heifers, and lactating cows. Differential KEGG functions on level 3 between growing heifers (**A**), heifers (**B**), and dairy cows (**C**). Significantly different CAZyme functions between heifers (**D**). (**F**) KEGG map of the TCA cycle pathway in heifers.

As shown in Fig. 4, heat stress decreased carbohydrate metabolism in growing heifers and heifers. Further analysis showed that heat stress caused a significant decrease in amino sugar and nucleotide sugar metabolism (ko00520), fructose and mannose metabolism (ko00051), and ascorbate and aldarate metabolism (ko00053) in the C-HS group (*P* < 0.05; Fig. 4A). Heat stress resulted in significantly lower starch and sucrose metabolism (ko00500), galactose metabolism (ko00052), fructose and mannose metabolism (ko00051), and ascorbate and aldarate metabolism (ko00053) in the H-HS group than that in the H-N group (*P* < 0.05; Fig. 4B). Only ribosomes (ko03008) were significantly increased by heat stress in lactating cows (*P* < 0.05; Fig. 4C). Seven different GH were selected in heifers, all of which were significantly reduced by heat stress (*P* < 0.05; Fig. 4D). The differential pathway citrate cycle (TCA cycle) (ko00020) in heifers increased significantly in the H-HS group (*P* < 0.05). Therefore, the genes that were enriched in the H-HS group were selected for the cluster heatmap (Fig. 4E).

### Serum metabolome and rumen metabolome

**Fig 5:**
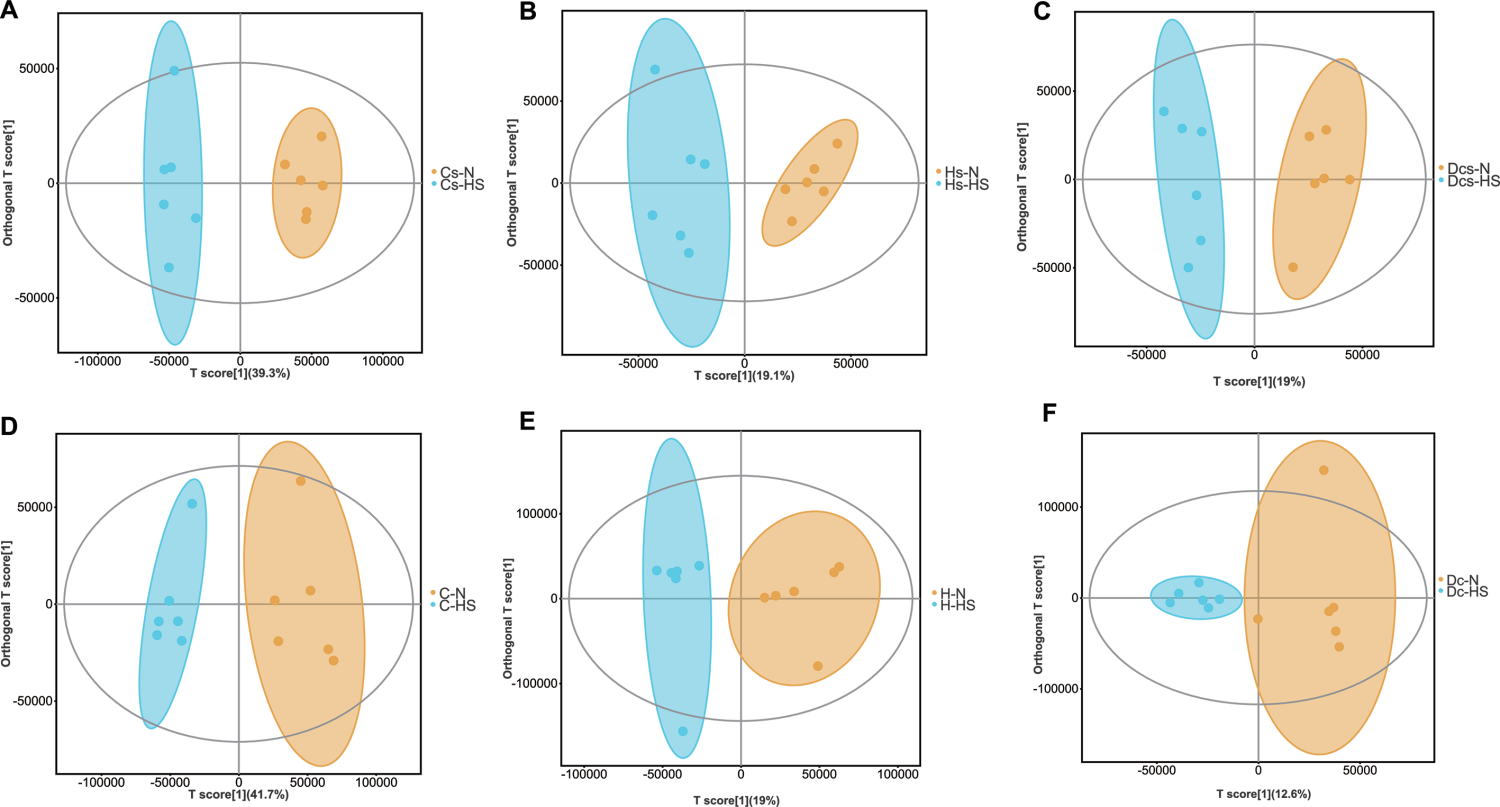
Orthogonal partial least squares discriminant analysis of growing heifers (**A**), heifers (**B**), and lactating cows (**C**) (positive and negative ion modes) in the ruminal fluid metabolome. OPLS-DA of growing heifers (**D**), heifers (**E**), and lactating cows (**F**) in the serum metabolome.

**Fig 6:**
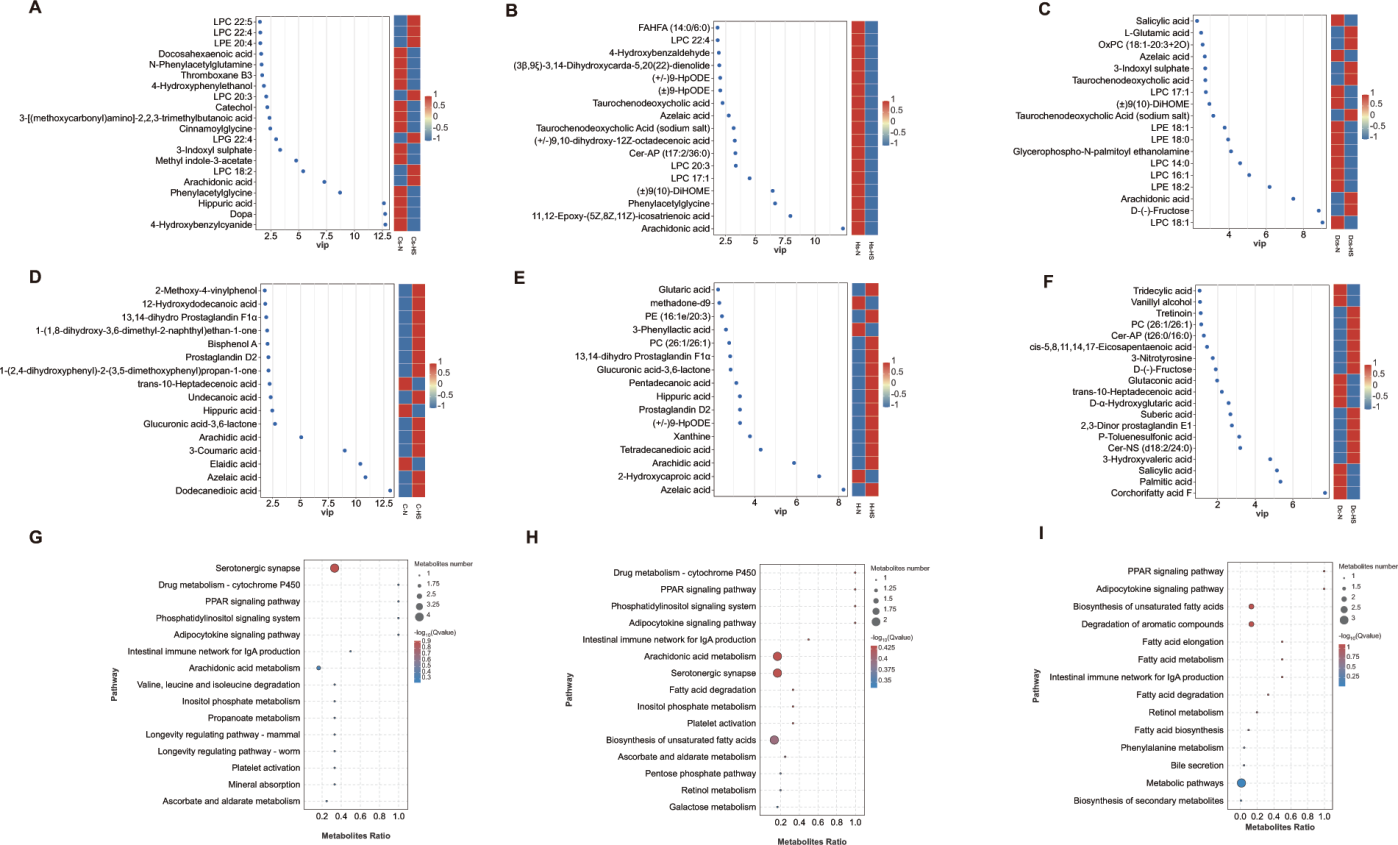
Metabolome of growing heifers, heifers, and lactating cows. Differential metabolite in the blood of growing heifers (**A**), heifers (**B**), and lactating cows (**C**). Differential metabolite in the rumen fluid of growing heifers (**D**), heifers (**E**), and lactating cows (**F**). Differential metabolic pathway enrichment bubble map in the rumen fluid among growing heifers (**G**), heifers (**H**), and lactating cows (**I**).

Liquid chromatography–mass spectrometry (LC-MS)-based metabolomic analysis of the serum and rumen fluid metabolites of cows at different growth stages revealed that when compared with cows in a normal climate, the rise in temperature changed the profile of the serum and rumen fluid metabolites of cows at different growth stages differently (Fig. 5). Identification of differential metabolites in Holstein cows at different growth stages was screened by using the VIP value (VIP > 1) of orthogonal partial least squares discriminant analysis (OPLS-DA) combined with t-test (P ≤ 0.05); in the Cs-N vs Cs-HS, the most important metabolites were 4-hydroxybenzylcyanide, Dopa, hippuric acid, phenylacetylglycine, and arachidonic acid (AA) (Fig. 6A); in Hs-N vs Hs-HS, the most important metabolites were arachidonic acid, 11,12-epoxy-(5Z, 8Z, 11Z)-icosatrienoic acid, and phenylacetylglycine (Fig. 6B). Among them, differential metabolites belonging to the fatty acyl group accounted for the largest proportion. Except for valproic acid, which was significantly enriched in the Hs-HS group, and the remaining nine fatty acyl metabolites were more enriched in Hs-N, such as arachidonic acid, azelaic acid, suberic acid, thromboxane B2, 16(R)-HETE, 9-Oxo-ODE, oleic acid, and palmitoleic acid; in Dcs-N vs Dcs-HS, several important metabolites were LPC 18:1, D-(-)-fructose, and arachidonic acid (Fig. 6C); in C-N vs C-HS, several important metabolites were dodecanedioic acid, azelaic acid, elaidic acid, and 3-coumaric acid. (Fig. 6D); in H-N vs H-HS, the important metabo­ lites were azelaic acid, 2-hydroxycaproic acid, and arachidic acid (Fig. 6E); in Dc-N vs Dc- HS, the important metabolites were corchorifatty acid F, palmitic acid, salicylic acid, and 3-hydroxyvaleric acid (Fig. 6F).

Through KEGG pathway analysis, we found that the PPAR signaling pathway was significantly enriched in growing heifers (Fig. 6), heifers (Fig. 6H), and lactating cows (Fig. 6I).

**Fig 7:**
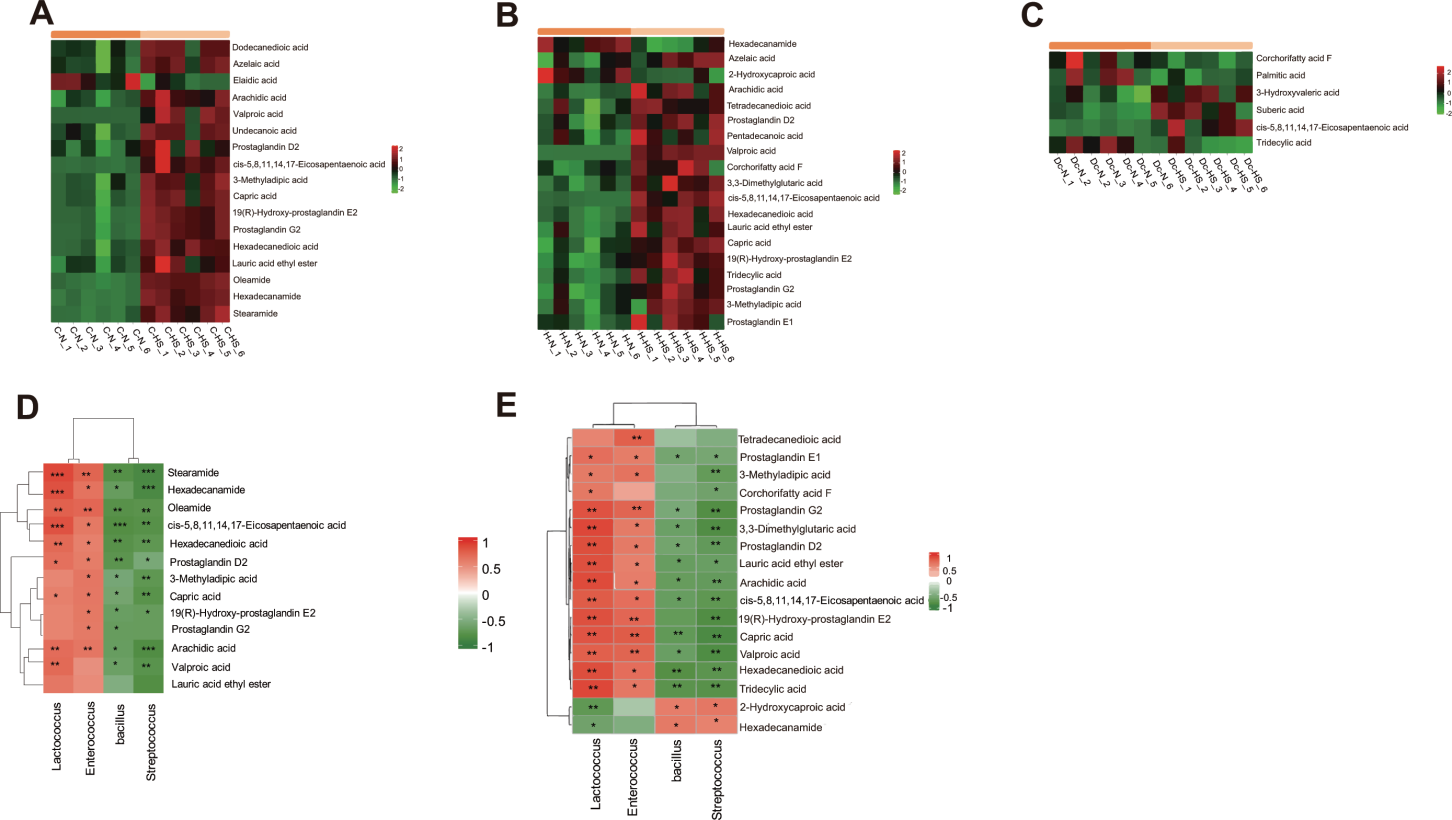
Enrichment analysis of differential fatty acyl metabolites in growing heifers (**A**), heifers (**B**), and lactating cows (**C**). Differential bacteria and differential acyl metabolites associated with growing heifers (**D**) and heifers (**E**).

C-N and C-HS identified 81 differential metabolites; the differential metabolites with the most proportion were the fatty acyl group. There were 16 enriched biomarkers in the C-HS group, dodecanedioic acid, azelaic acid, arachidic acid, valproic acid, undecanoic acid, prostaglandin (PG) D2, cis-5, 8,11, 14, 17-eicosapentaenoic acid, 3-methyladipic acid, capric acid, 19(R)-hydroxy-prostaglandin E2, prostaglandin G2, hexadecanedioic acid, lauric acid ethyl ester, oleamide, hexadecanamide, and stearamide; only elaidic acid was more enriched in the C-N group (Fig. 7A).

A total of 64 differential metabolites were identified in the H-N and H-HS groups, and 55 differential metabolites were significantly enriched in the H-HS group (*P* < 0.05), among which fatty acyl differential metabolites accounted for the largest proportion. Except 2-hydroxycaproic acid and hexadecanamide that were significantly enriched in H-N group, the remaining 17 fatty acyl metabolites, such as azelaic acid, arachidic acid, tetradecanedioic acid, prostaglandin D2, pentadecanoic acid, valproic acid, corchorifatty acid F, 3,3-dimethylglutaric acid, cis-5,8,11,14,17-eicosapentaenoic acid, hexadecanedioic acid, lauric acid ethyl ester, capric acid, 19(R)-hydroxy-prostaglandin E2, tridecylic acid, 3-methyladipic acid, prostaglandin E1, and prostaglandin G2, were highly enriched in H-HS (Fig. 7B). Moreover, some carboxylic acids and derivatives, such as glutaric acid, gamma-glutamylglutamine, and proline hydroxyproline, were highly abundant in the H-HS group.

Twenty differential metabolites were identified between the Dc-N and Dc-HS groups. The differential metabolites with the highest proportion were fatty acyl; the biomarkers with six metabolites belonged to fatty acyl. Three biomarkers in the Dc-HS group were 3-hydroxyvaleric acid, suberic acid, and cis-5,8,11,14,17-eicosapentaenoic acid, and only corchorifatty acid F, tridecylic acid, and palmitic acid were enriched in the Dc-N group (Fig. 7C). Spearman’s association analysis revealed that Lactococcus, Enterococcus, Bacillus, and Streptococcus were significantly associated with differential fatty acyl metabolites in growing heifers and heifers (Fig. 7D and E).

**Fig 8:**
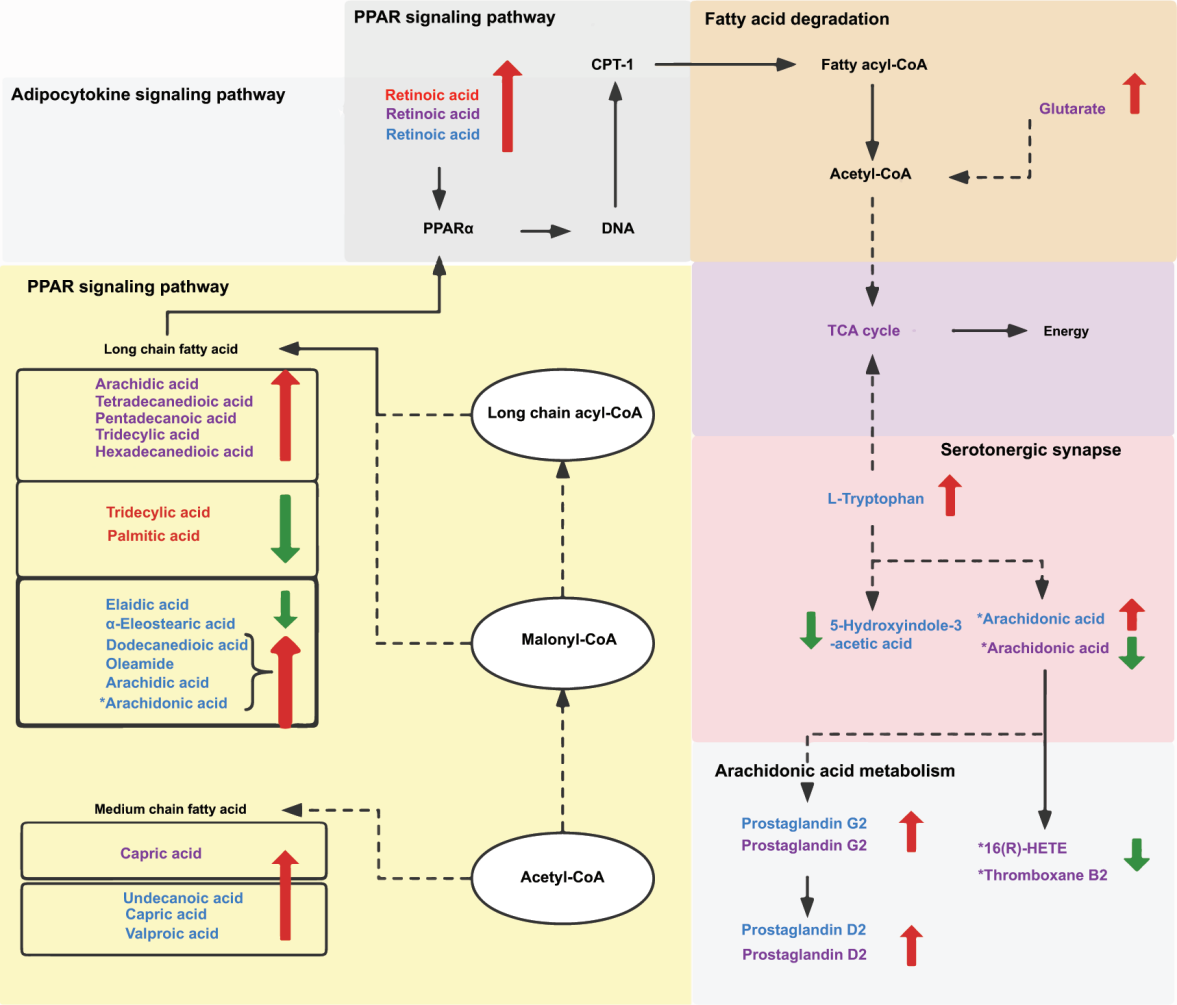
Overview of the experimental results. The blue characters represent growing heifers. The purple character represents the heifers. The red characters represent lactating cows. Red arrows represent the upregulation of this metabolite due to heat stress. Green arrows represent the downregulation of this metabolite due to heat stress. * indicates that the metabolite is a biomarker screened in serum. Not labeled indicates that the metabolite is a biomarker screened in rumen fluid.

In summary, our study involved the integration of biomarkers derived from the long-chain fatty acids (FAs), including arachidic acid and palmitic acid, as well as medium chain fatty acids, arachidonic acid, prostaglandins, and others. We found that they were collectively involved in various metabolic pathways such as arachidonic acid metabolism, PPAR signaling pathway, adipocytokine signaling pathway, and other metabolic pathways (Fig. 8).

## DISCUSSION

The risk of metabolic disorders increases owing to heat stress in dairy cows; however, the mechanisms underlying the metabolic changes resulting from heat stress remain unclear. In this study, we used integrated multiomics data to analyze the correlations among rumen microbiota, metabolites, and host phenotypes in heat-stressed dairy cows. The results showed that heat stress, except in growing heifers, did not significantly affect heifers or lactating cows and rumen microbial diversity was relatively stable when ruminants approached adulthood (15). Cattle in the C-HS group had higher OTU counts and alpha diversity (Chao1, sob, and ACE), indicating that the species richness of the ruminal fluid microbiota increased owing to heat stress. Heat stress leads to increased abundance of pathogenic bacteria in cattle herds at each growth stage, such as the C-HS group increased the abundance of *Treponema_2* and the H-HS group increased the abundance of pathogenic bacteria, such as *Erysipelotrichaceae _ UCG-004* and *Treponema_2*, which is consistent with previous studies (11). Heat stress causes decreased abundance of beneficial bacteria in lactating cows, such as *Bifidobacterium*, *Acetobacteraceae*, and *Lactobacillus*. Increased *Sphingobacterium* (16) and *Streptococcus* (17) in lactating cows were associated with increased somatic cell count (SCC). Previous studies have shown that the rumen of dairy cows does not produce fiber-degrading enzymes to decompose ingested fiber substances but relies on the degradation and digestion of microorganisms, such as bacteria and fungi (18). The main physiological function of *Firmicutes* is the degradation of structural polysaccharides (19), which contains a large amount of fiber decomposition of bacteria (20), and *Bacteroidetes* is the main degrader of non-fibrous carbohydrates of ruminal microorganisms, contains genes related to the degradation of non-fibrous polysaccharides (21), and may promote the host uptake of monosaccharides. In addition, *Prevotella_1* in *Bacteroidetes* was the dominant genus in growing heifers, heifers, and lactating cows, and the relative abundance of *Prevotella_1* in the rumen increased with increasing age. However, according to the analysis of growing heifers, heifers, and lactating cows at different growth stages, we found that the abundance of *Firmicutes*, *Ruminococcus_2*, *Bacteroidetes*, and *Prevotella* increased in both growing heifers and heifers and were relatively stable in lactating cows. The abundance of *Bacteroidetes* and *Prevotella* increased with age, indicating that the efficiency of carbohydrate decomposition may increase with age. *Prevotella* species are associated with a plant-rich diet rich in carbohydrates and fiber (22). *Prevotella* has the potential to digest and break down complex carbohydrates from food, sugars, and cellulose (23).

*Clostridium* has a high cellulose-degrading activity, actively generates hydrogen (24), and uses butyrate kinase instead of coenzyme A transferase in the final step of butyrate formation (25). Heat stress significantly decreased the relative abundance of *Clostridium_sensu_stricto_3* in growing heifers and heifers. These results suggest that carbohydrate metabolism was reduced in growing heifers and heifers, possibly due to changes in cellulolytic microbiota during heat stress (Fig. 3). Functional analysis of metagenomes provided further evidence that heat stress led to a significant reduction in carbohydrate metabolism in growing heifers and heifers and that heat stress significantly affected the balance between carbohydrate and energy metabolism. From the perspective of the rumen microbial function differential pathway, the carbohydrate metabolism of the C-N and H-N group cows was abundant, including ko00053 (ascorbate and aldarate metabolism) and ko00051 (fructose and mannose metabolism), indicating that heat stress reduced the carbohydrate degradation capacity of the C-HS group and H-HS cows. Carbohydrate-active enzymes are a series of enzymes capable of degrading, modifying, and generating glycosidic bonds (26). The ability of ruminants to decompose plant biomass into fermentable sugars can be entirely attributed to polysaccharide hydrolases produced by the rumen microbiota (27). The CAZy database provides information on all CAZyme families, GHs, GTs, PLs, CEs, CBMs, and AAs (28), and our data show that GHs are the most abundant enzymes in the H-N group compared with those in the H-HS group (29, 30). To obtain further information about the GH families, we classified the families to which the GHs belong to according to Krause et al. (31). Numerous genes encoding β-glucosidase, β-xylosidase, and cellulose endonuclease were highly enriched in the rumen microbiota of the H-N group. GH is responsible for promoting the hydrolysis of cellulose (32). In the present study, enzymes of the GH family in the rumen metagenome of the H-N group exhibited a complex cellulose degradation process. Combined with the enrichment of genes (GHs) involved in carbohydrate decomposition in the rumen microbiota of dairy cows in the H-N group, we further demonstrated that heifers in the H-N group had high ability to degrade complex substrates. Ascorbic acid and aldose metabolism are indirectly associated with biosynthetic pathways with significant antioxidant potential (33). Significant enrichment in the H-N group indicated a high antioxidant capacity, while the oxidative phosphorylation pathway showed a significant enrichment of the oxidative phosphorylation pathway in the H-HS group.

The composition of rumen microorganisms varies with time, environment, season, and host genetic factors (34); however, the possibility that the function of the microbial community is changed by these factors is conservative. The function of the rumen microbiota is taxonomically conserved. Heat stress in lactating cows had no significant effect on rumen KEGG differential genes; only the translation function (Ko03010) in genetic information processing was affected, which may be related to the different growth stages of Holstein cows. By comparing the different functions of cows at different growth stages, we found that heat stress caused differences in the functions of Holstein cows; however, the degree of influence of heat stress at different growth stages was different, and functional changes were highly conservative with an increase in age. Changes in microbial function are not only more conserved than changes in microbial composition but are also affected by age. *Lactobacillus* is associated with lipid metabolism through its role in bile salt biotransformation (35). Heat stress results in an increased relative abundance of *Bacillus*, *Lactococcus*, *Lactobacillus*, *Streptococcus*, and *Enterococcus* in heifers. The relative abundance of *Lactobacillus_concavus_DSM_17758* and *Enterococcus* in growing heifers increased, but the abundance of *Bacillus* and *Streptococcus* decreased. These bacteria are associated with bile brine hydrolysis enzyme (BSH) activity (36). Moreover, heat stress leads to high enrichment of differential fatty acyl metabolites in growing heifers, heifers, and lactating cows in metabolomic analysis of rumen fluid. Therefore, correlation analysis between five BSH-related microorganisms in growing heifers, heifers, and differences in acyl metabolites showed that there was no significant difference in *Lactobacillus*, but *Lactococcus*, *Bacillus*, *Streptococcus*, and *Enterococcus* were associated with significant fatty acyl compounds. The H-HS and C-HS group cholesterol steady equilibrium may be destroyed, and further promotion of non-alcoholic fatty liver disease (NAFLD) by influencing the FXR-FGF15/19 signaling pathway (37, 38) requires further detection. A variety of differential metabolites were detected between C-N and C-HS, H-N and H-HS, and Dc-N and Dc-HS, which may be related to the nutritional metabolism mechanism of dairy cows in different seasons, indicating that the identification of differential metabolites may be helpful in assessing physiological conditions, such as heat stress (39). Metabolites in the rumen fluid are a direct and effective method for evaluating the metabolic characteristics of dairy cows under heat stress, and the serum is a comprehensive reflection of dairy cow metabolism. In our study, overlapping differential metabolites in the serum and rumen fluid were integrated as important indicators for heat stress screening in growing heifers, heifers, and lactating cows (40). We integrated the serum and rumen fluid metabolomes and screened four common metabolites, hippuric acid, 4-hydroxybenzaldehyde, 3-bromo-2,6-dimethoxybenzoic acid, and L-tryptophan (L-Trp), in the serum and rumen fluids of growing heifers. Among these, hippuric acid and L-Trp are derived from a common metabolic pathway between the host and rumen microbiota. Heat stress resulted in a decrease in hippuric acid and an increase in L-Trp in the blood and rumen fluid. L-Trp is an essential amino acid (41), and L-Trp has been shown to reduce oxidative stress and immune suppression (42). L-Trp alleviates oxidative damage in ruminants (43). Although no changes in glycine were detected in the differential metabolites, a variety of acylglycines (hippuric acid and phenylacetyl glycine) were found in the serum of growing heifers, and hippuric acid was significantly upregulated in the rumen fluid of growing heifers. Among these significantly changed acylglycine, hippuric acid is benzoylglycine, a conjugate of benzoic acid and glycine that is synthesized mainly in the liver, through which the body eliminates the toxicity of benzoic acid. Therefore, the significant upregulation of hippuric acid concentration in the Cs-N and C-N groups indicates that benzoic acid metabolism may be affected, which contributes to the metabolism of benzoic acid and other metabolic wastes in the body and reduces the risk of disease (44).

Six common metabolites were screened in heifers serum and rumen fluid, including valproic acid, cyclohexaneacetic acid, azelaic acid, (±)9-HpODE, (±)9-HpODE, and 2,3-dinor prostaglandin E1. We screened six common metabolites in serum and rumen fluid of lactating cattle, including D-(-)-fructose, salicylic acid, 2,3-dinor prostaglandin E1, PC (26:1/26:1), corchorifatty acid F, and suberic acid. Suberic acid, azelaic acid, and pimelic acids are involved in lipid transport and metabolism, fatty acid metabolism, and lipid peroxidation, respectively (45). This indicated that fatty acid peroxidation disorders occur in both heifers and lactating cows. Among them, salicylic acid and D-(-)-fructose are differential metabolites derived from the rumen microbiota, which have also been found in previous studies (46). Changes in salicylic acid content were also detected in left displaced abomasum cows, which may be related to changes in taurochenodeoxycholic acid (TCDCA). This shows that the changes in salicylic acid may represent a possible lipid metabolism disorder in dairy cows under heat stress; however, its relationship with TCDCA requires further study. TCDCA levels were significantly downregulated in the Dcs-HS and Hs-HS groups. TCDCA is a conjugated bile acid with a significant anti-inflammatory effect (47) and suppresses prostaglandins in inflammatory tissues (48), indicating that the antioxidant effect on lactating cows and heifers decreases during heat stress. 3-Indolylsulfate was also detected in the Dcs-HS group, which is a host-microbiome co-metabolite (49). The Dc-HS group significantly upregulated the expression of 3-nitrotyrosine, an oxidation product of tyrosine, which further reduces the antioxidant capacity of the body, leading to increased intracellular oxidation.

Arachidonic acid and its derivatives link nutrient metabolism to immunity and inflammation and thus play a key role in the development of NAFLD (50, 51). Under heat stress, AA metabolism is significantly enriched in the rumen fluid metabolites of heifers, which can be converted into a series of PGs mediated by cyclooxygenases (COXs). COX 1 and COX 2, also known as prostaglandin G/H synthases, promote the production of prostacyclin (PGI 2) and several PGS. PG D2 is a metabolite of the COX 1 pathway. PGs are important lipid mediators that regulate immunity and inflammation (52, 53). Long-chain fatty acids activate the ligand PPARs (54, 55), and PPAR α regulate lipid metabolism in the liver (56), which mainly controls systemic nutrition and energy balance, whose abnormalities may lead to steatohepatitis and fatty fibrosis. The degradation of fatty acids or β-oxidation is primarily regulated by PPAR α. Constitutive mitochondrial β-oxidation activity is significantly reduced in the livers of mice lacking the PPAR α gene (PPAR-deleted mice) (57). When PPAR α is activated by FA, the β-oxidation and adenosine triphosphate are enhanced, indicating that PPARα is the main control factor of FA oxidation and energy production under heat stress.

## MATERIALS AND METHODS

### Management and sample collection of Holstein cows

In this experiment, Shandong High Speed Dairy Co. Ltd. provided the dairy cows. Healthy growing heifers (6.93 ± 0.14 months of age, and *n* = 10), heifers (16.54 ± 0.11 months of age, and *n* = 10), and lactating cows with two litters (41.02 ± 0.36 months of age, and *n* = 10) of similar body condition were separately selected. The test was divided into two stages: heat stress period and normal period. The normal group of growing heifers, heifers, and lactating cows were sampled during the natural conditions of April (THI mean = 50.76), while the heat stress group was sampled in August (THI mean = 81.11). Importantly, it was the same batch of cows sampled under different temperatures, resulting in a total of 60 samples collected.

Then, the cattle were divided into six treatment groups based on the herd growth stage and THI: growing heifers normal group (C-N), growing heifers heat stress group (C-HS), heifers normal group (H-N), heifers heat stress group (H-HS), lactating cows normal group (Dc-N), and lactating cows heat stress group (Dc-HS). Cows experienced normal phenomena and heat stress more than 3 weeks before sampling. The THI was calculated as follows (58):


$$THI=0.8\times ambient\;temperature+\frac{\%relative\;humidity}{100}\times (ambient\;temperature-14.4)+46.4.$$

THI-related data were recorded thrice per day (at 06:00, 14:00, and 22:00), and the average of the three measurements was used as the THI for each day. THIs of the normal and heat-stressed groups have been reported (14). Rectal temperature and respiratory rate were measured thrice per day (06:00, 14:00, and 22:00). Rectal temperature was measured using a veterinary thermometer, and the respiratory rate was determined by counting the number of lateral abdominal movements in cows within 1 min. Rumen fluid samples were collected in oral stomach tubes by squeezing and filtering through three layers of gauze.

Blood (10 mL) was collected from the caudal root vein of each cow and placed in a pro-coagulant tube. The blood was centrifuged at 3,500 r/min for 10 min after 30 min at room temperature, and the supernatant was divided into 1.5-mL centrifuge tubes. To avoid variation, the separated serum samples were immediately stored in a refrigerator at −80°C for blood markers and metabolomics.

### 16S rDNA sequencing in the rumen fluid of Holstein cows

Samples of rumen fluid were collected, immediately frozen in liquid nitrogen, and stored at −80°C. Total DNA was extracted from samples using the HiPure Stool DNA Kit (Magen, Guangzhou, China). The quantity and quality of the purified DNA were for 16S rDNA and metagenomic sequencing using a NanoDrop spectrophotometer (Thermo Fisher Scientific, Waltham, MA, USA), gel electrophoresis, and a gel imaging system. DNA quality was determined using a Qubit (Thermo Fisher Scientific, Waltham, MA, USA) and NanoDrop (Thermo Fisher Scientific, Waltham, MA).

Targeted 16S sequencing libraries were prepared according to the 16S metagenomic sequencing library preparation protocol (Illumina, San Diego, CA, USA) using sequencing primers and sequencing adapters. The protocol included two primers that selectively amplified the V3–V4 region-specific region (341F:CCTACGGGNGGCWGCAG; 806R: GGACTACHVGGGTATCTAAT) of 16S rDNA. The concentrations of the 16S rDNA libraries were assessed using an Agilent 2100 bioanalyzer (Agilent DNA 1000 Reagents) and a Genomic DNA Sample Prep Kit for the Illumina Nova6000 Platform.

Amplicons extracted from 2% agarose gels were purified using an AxyPrep DNA Gel Extraction Kit (Axygen Biosciences, Union City, CA, USA) and quantified using an ABI StepOnePlus Real-Time PCR System (Life Technologies, Foster City, USA) according to the manufacturer’s instructions. Purified amplicons were pooled in equimolar amounts and sequenced (PE250) on an Illumina platform according to the standard protocols of the NovaSeq 6000 sequencing platform (Illumina, San Diego, CA, USA) using paired-end technology and single-ended indices, as recommended by the manufacturer.

Raw reads were filtered and trimmed to eliminate low-quality bases and adaptor sequences according to the following rules using FASTP (version 0.18.0)(59):

1. Reads containing >10% unknown N were removed.
2. Reads containing <50% bases with quality (*Q*-value) > 20 were removed.

Clean tags were clustered into OTUs based on  ≥97% similarity using the UPARSE (version 9.2.64) (60) pipeline. Weighted and unweighted UniFrac distance matrices were generated using the GuniFrac package (version 1.0) (61) in Jaccard. The Sob, Chao1, ACE, Shannon, and Simpson indices were calculated using the Python scikit-bio package (version 0.5.6) (62). Alpha index comparison between groups was performed using Welch’s *t*-test and the Wilcoxon rank test in the R Project Vegan Package (62).

### Construction of the sequencing libraries and the metagenomic sequencing

Total DNA was extracted, and its quality was tested and found to be consistent with that of 16S rDNA. Metagenomic libraries were constructed using a Genomic DNA Sample Prep Kit (Illumina, San Diego, CA, USA), following the manufacturer’s recommendations. Qualified genomic DNA was fragmented via sonication to a size of 350 bp, and then end repaired, A tailed, and adaptor ligated using the NEBNext ΜLtra DNA Library Prep Kit for Illumina (NEB, USA), according to the preparation protocol. DNA fragments with lengths of 300–400 bp were amplified using PCR. Finally, the PCR products were purified using the AMPure XP System (Beckman Coulter, Brea, CA, USA), and the libraries were analyzed for size distribution using a 2100 Bioanalyzer (Agilent, Santa Clara, CA, USA) and quantified using real-time PCR. Genome sequencing was performed using the Illumina NovaSeq 6000 sequencer with paired-end technology (PE 150).

### Metabolomics analysis of LC-MS

Rumen fluid and blood were collected from Holstein cows for untargeted metabolomic analysis via LC-MS. Serum (100 µL) was placed in EP tubes (Eppendorf tubes) and resuspended in 400 µL of pre-chilled 80% methanol via thorough vortexing. Then, the samples were incubated on ice for 5 min and centrifuged at 15,000 × *g* and 4°C for 20 min. Some of the supernatant was diluted to a final concentration containing 53% methanol with LC-MS-grade water. The samples were subsequently transferred to fresh Eppendorf tubes and then centrifuged at 15,000 × *g* and 4°C for 20 min. Finally, the supernatant was injected into an LC-MS/MS system for analysis (63, 64). A Vanquish UHPLC System (Thermo Fisher, Germany) coupled with an Orbitrap Q Exactive HF-X Mass Spectrometer (Thermo Fisher, Germany) was used for UHPLC-MS/MS analyses. Samples were injected onto a Hypesil Gold column (100 × 2.1 mm, 1.9 µm) using a 17-min linear gradient at a flow rate of 0.2 mL/min. Eluents A (0.1% FA in water) and B (methanol) were used for the positive polarity mode. The eluents used for the negative polarity mode were eluents A (5 mM ammonium acetate, pH 9.0) and B (methanol). The solvent gradient was set as follows: 2% B, 1.5 min; 2–100% B, 12.0 min; 100% B, 14.0 min; 100–2% B, 14.1 min; and 2% B, 17 min. Q ExactiveTM HF-X mass spectrometer was operated in positive/negative polarity mode with a spray voltage of 3.2 kV, capillary temperature of 320°C, sheath gas flow rate of 40 arb, and auxiliary gas flow rate of 10 arb.

### Statistical analysis

Multivariate statistical techniques, including PCoA and non-metric multidimensional scaling of Bray-Curtis distances, were calculated using the R vegan package and plotted using the R ggplot2 package (65). The abundance statistics for each taxon were visualized using Krona (version 2.6) (66). Venn analysis was performed using the R project VennDiagram package (67); Biomarker features of species and functions in each group were screened using LEfSe software (version 1.0) .

Function comparison between groups was performed using Welch’s *t*-test in the R project Vegan package. Differentially enriched KEGG pathways were identified according to their reporter scores from the Z-scores of the individual KEGG Orthologs (Kos).

In the subsequent data analysis, the positive and negative ion modes were analyzed separately, and OPLS-DA was applied using R package models (http://www.r-project.org/). The OPLS-DA model was further validated using cross-validation and permutation tests (68). The VIP score of the OPLS model was used to rank the metabolites that best distinguished the two groups. The threshold of the VIP was set to 1. In addition, the *t*-test was also used as a univariate analysis for screening differential metabolites. Those with a *P* value of *t*-test＜0.05 and VIP ≥ 1 were considered differential metabolites between two groups. A correlation heat map was constructed using the R Corrplot package (69). The MetOrigin platform (70) was used to analyze the association between microbiota and differential metabolites.

## Data Availability

The raw data for each sample of 16S rDNA, metagenomic sequencing, and metabolomics have been reported previously (14). The sequencing data of 16S rDNA and metagenome in this study have been submitted to the NCBI Sequence Read Archive (SRA) database (https://www.ncbi.nlm.nih.gov/sra), and we have obtained the following BioProject accession numbers: PRJNA850536 (71) and PRJNA850514 (72). The raw LC-MS data files of rumen fuid samples and serum samples were converted to.mgf format by using ProteoWizard package (http://proteowizard.sourceforge.net), were deposited, and are publicly available at the MetaboLights database (73) (http://www.ebi.ac.uk/metabolights) of the European Bioinformatics Institute under MTBLS5132 (74) and MTBLS5148 (75).

## SUPPLEMENTAL MATERIAL

Fig S1 spectrum.03312-23-s0001.pdf ANOSIM analysis.

ASM does not own the copyrights to Supplemental Material that may be linked to, or accessed through, an article. The authors have granted ASM a non-exclusive, world-wide license to publish the Supplemental Material files. Please contact the corresponding author directly for reuse.
