# Supplementary material for: Altered rumen microbiome and correlations of the metabolome in heat-stressed dairy cows at different growth stages
Source: Microbiol Spectr. 2023 Nov 16;11(6):e03312-23. doi: 10.1128/spectrum.03312-23 (PMC10714726; doi:10.1128/spectrum.03312-23)
Supplement: Fig S1 — ANOSIM analysis. [file spectrum.03312-23-s0001.pdf]

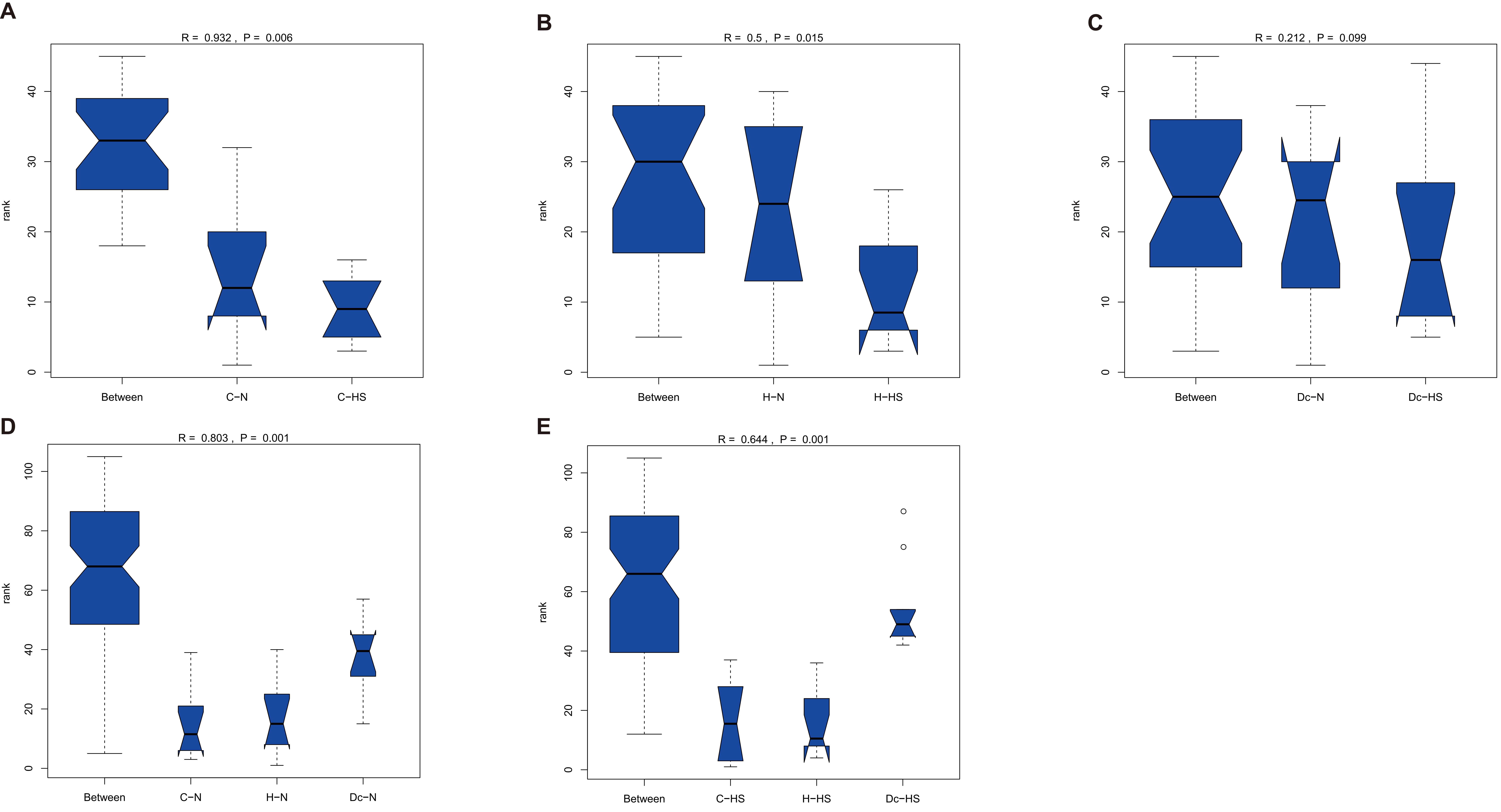

FIG S1 ANOSIM analysis. ANOSIM analysis in the growing heifers (A), heifers (B), and lactating cows (C); ANOSIM analysis in the C-N, H-N, and Dc-N (D); ANOSIM analysis in the C-HS, H-HS, and Dc-HS (E).
